# Growth, survivorship, and predator avoidance capability of larval shortnose sturgeon (*Acipenser brevirostrum*) in response to delayed feeding

**DOI:** 10.1371/journal.pone.0247768

**Published:** 2021-03-17

**Authors:** Ryan S. Hardy, Vahid Zadmajid, Ian A. E. Butts, Matthew K. Litvak

**Affiliations:** 1 Department of Biology and Centre for Coastal Studies and Aquaculture, University of New Brunswick, Saint John, Canada; 2 Idaho Department of Fish and Game, Coeur d’ Alene, Idaho, United States of America; 3 Department of Fisheries Science, Faculty of Natural Resources, University of Kurdistan, Sanandaj, Iran; 4 School of Fisheries, Aquaculture and Aquatic Sciences, Auburn University, Auburn, Alabama, United States of America; 5 Department of Biology, Mount Allison University, Sackville, New Brunswick, Canada; Tanzania Fisheries Research Institute, UNITED REPUBLIC OF TANZANIA

## Abstract

Larval shortnose sturgeon, reared at 17°C, were subjected to delayed feeding treatments of 0, 5, 10, 15, 18, and 23 days post-yolk absorption to examine effects of food deprivation on growth, survival, swimming activity, and escape capabilities. Starvation affected growth and survival but despite degree of starvation, larvae were able to resume growth and experience high survivorship following feeding. Specific growth rate based on larval dry weight for the period directly following first feeding was highest for the day 15 and 18 delayed feeding treatments. There were no differences in survival between the 0 and 5 day treatments, however survival was reduced to 71.2%, 45.4%, and 28.8% for 10, 15, and 18 day delayed feeding treatments, respectively. Shortnose sturgeon had a point-of-no-return (PNR; 55.7% initiated feeding) at ~19 days (or 42 days post-fertilization) following the full absorption of yolk. Mean percent swimming activity and swimming speeds showed an interaction between delayed feeding treatment and larval age, such that no differences were detected at 1 and 6 days post-yolk absorption, while these swimming behaviors generally increased or spiked as feeding was delayed for 10, 15, and 18 days post-yolk absorption. At 23 days post-yolk absorption, only swimming speed increased for larvae that were denied food for 18 days. While there was an interaction between delayed feeding treatments and age for proportion of larvae exhibiting an escape response, generally, larvae from all feeding treatments exhibited a positive escape response. There were also interactions between delayed feeding treatments and age post-yolk absorption for mean and maximum escape speeds, such that less aggressive escape responses were typically detected the longer larvae were denied food. Our research suggests that larval shortnose sturgeon increase physical activity during periods of starvation to find a food patch while remaining vigilant but maybe not as capable to defend against a predatory attack as fed individuals.

## Introduction

The ability of fish larvae to grow and survive through their early life history (ELH) stages ultimately plays a role in recruitment and year-class formation [1, 2]. Starvation and predation during these ELH stages have long been hypothesized as being the primary factors involved in regulating survival [1, 3–5]. Larval susceptibility to predation, linked to such factors as frequency of encounter and ability to avoid and escape an attacking predator, may be affected by the degree of starvation [6, 7]. Examining the interaction between starvation and predation and how it affects larval survival is important to our understanding of recruitment.

Predation is considered to be the main cause of mortality during the egg and yolk-sac stage [1, 8], while direct mortality due to starvation only occurs following the transition to exogenous feeding [9]. Linked to patchy or inadequate distribution of food resources prior to the initiation of feeding, this “critical period” where there is a switch to exogenous food sources has been suggested to be a period of increased mortality [3, 10]. Cushing extended Hjort’s critical period hypothesis with the “match-mismatch” hypothesis [11]. Cushing [11] hypothesized that larval survival and recruitment is conditioned by the match of larvae with prey fields in time and space. Therefore, larvae must locate food patches during this critical period before a time of irreversible starvation or “point-of-no-return (PNR)” is reached. PNR is defined as the point during starvation where 50% of the larvae are still alive yet are unable to feed even when food becomes available [12].

Some species of larval fish found in “mismatch” conditions are able to resist and recover from the effects of short periods (days) of low food [13] or starvation [14]. However, during this time, starvation can result in increased vulnerability to predation. For example, starving larvae may be less able to avoid and escape attacking predators than fed larvae [6, 15]. Alternatively, smaller size and reduced activity during starvation may reduce the likelihood of being located by predators [9]. It has been suggested that temporal variation in risks to predation may be a crucial element dictating animal behavior [16, 17]. Therefore, a starving larva may use different behaviors to increase chances of locating food while simultaneously remaining vigilant and responsive to predators. There are two schools of thought on the most probable response, similar to the “risk allocation” hypothesis [16], to starvation by a developing larva: 1) a larva will reduce activity to conserve energy for predator alertness and forage once a food patch is encountered [18]; and 2) in spite of the energetic costs, a larva maintains risk responsiveness and raises activity levels to increase the probability of encountering a food patch [7]. Regardless of the strategy used, increased vulnerability of larvae during mismatch conditions, compounded with slow growth and development, may ultimately result in increased mortality during this larval stage.

Information is somewhat limited on the effects of starvation on susceptibility of fish larvae to predation and has mainly focused on marine species [7, 19–21]. Early-life mortality in sturgeon is not well researched or understood. Yet this stage is particularly important in determining year-class strength since their large body size, tough leathery skin, and bony scutes translate to lower mortality risks during the juvenile and adult stages [22]. Elasticity analysis suggests that for three species of North American sturgeon, population dynamics are most sensitive to changes in survival during the first year of development [23]. The importance of examining such ELH mortality in sturgeon is even greater since many of these species are threatened or endangered throughout their range of distribution [24–26]. One such North American species, once commercially harvested, is the shortnose sturgeon, *Acipenser brevirostrum*.

Shortnose sturgeon are found in many of the large coastal waterways of eastern North America, ranging as far north as the Saint John River in New Brunswick and as far south as the St. John’s River in Florida [27–30]. Shortnose sturgeon are considered an amphidromous species that make small seasonal migrations from brackish to freshwater. Spawning is infrequent, with males spawning every 1–3 years and females every 3–5 years [27, 28]. Shortnose sturgeon spawn early in the spring at low temperatures [27, 28]. Once embryos absorb their yolk-sac, they experience a rapid change in behavior. Yolk-sac larvae of shortnose sturgeon from the Hudson and Connecticut rivers are photonegative and seek cover but switch to vertical migratory behavior after yolk-sac absorption [24]. Shortnose sturgeon larvae from the Saint John River also appear to engage in an active/passive migration during their early life history stages [31, 32]. They drift mainly at night and unlike that reported by Kynard and Horgan [24] will do so during the yolk-sac stages.

Foraging behavior of wild shortnose sturgeon larvae is not known, however, larvae reared in an artificial stream foraged on open sand substrate [24]. Starvation may be an important factor in determining survivorship since losses from starvation and predation are often size dependent [33, 34‏]. Usvyatsov et al. [31, 32] suggested that the substantial reduction in yolk-sac larval abundances, combined with the short period of migration between transects in their field study, was due to predation. Unfortunately, little is known about factors of mortality (i.e. natural predators or potential for starvation) and behavior of shortnose sturgeon larvae. Therefore, factors affecting their survival during this period are important to long term survival and subsequent recruitment. Due to severe population declines in eastern North America, shortnose sturgeon are now protected in the United States under the Endangered Species Act and considered a species of special concern in Canada [28]. Identifying strategies utilized by shortnose sturgeon to survive during low food availability may provide a better understanding of why this species is declining throughout most of its distribution.

Our study was conducted under simplified conditions that were designed to test the hypothesis that delayed feeding would affect shortnose sturgeon larval performance. Our first objective was to examine growth and starvation resistance of larval shortnose sturgeon in response to delayed feeding. Although two strategies for combining anti-predator/risk-responsiveness and activity level have been suggested, there are actually four possible combinations of these couplets. Thus, the second objective was to examine swimming activity and escape capabilities to determine which of the following responses or combination of responses are utilized during starvation: 1) reduction in activity levels and maintenance of high-risk responsiveness while waiting for a food patch; 2) increase in activity levels to locate a food source while maintaining high responsiveness to predators; 3) reduction in activity levels as well as responsiveness to predators to conserve energy; or 4) increase in activity levels to find a food patch yet making a trade-off by lowering risk responsiveness.

## Materials & methods

### Egg collection and incubation

Short-set gill nets were used to collect reproductively mature shortnose sturgeon in the Saint John River, New Brunswick, Canada (N 45°33’ W 66°02’; water temperature: 15–16°C) and held at Canadian Caviar’s sturgeon facility in Saint John, New Brunswick. Fertilization procedures (as specified by Doroshov et al. [35]) were performed using eggs collected from one female and sperm pooled from two mature shortnose sturgeon males. Fertilized eggs were incubated in MacDonald incubation jars (~500 mL in each) in a partial re-circulation system (3 L/min) at the University of New Brunswick, Saint John Campus. All eggs were incubated at a constant temperature of 17°C until hatching (~200 h post-fertilization).

### Egg size

It is possible that different size females as well as seasonal variations such as age, weight, and health can all play a role in egg size. In addition, it is true that egg size plays a role in the PNR of a larva and how long it can survive during complete starvation [36]. To make certain that the eggs from the single female cross were similar in size to that of other crosses, egg sizes from 4 independent shortnose sturgeon crosses and from two spawning seasons were compared using a two-way ANOVA. The resulting ANOVA showed no significant difference between egg size range within or between years (*P* = 0.134; 3.5 mm ± 0.088 SE following hydration).

### Experimental design

Directly following hatch (0 days post hatch [dph]), larvae were separated into six delayed feeding treatments, each of which contained three static replicate trays (1000 mL Pyrex trays [15 cm x 25 cm] filled with 700 mL of de-chlorinated water) stocked with 30 larvae. In the first treatment, food was offered to the larvae directly following yolk absorption, which was at 15 dph (termed, 0 day delayed feeding treatment). Larvae in the second through fifth treatments were starved for 5, 10, 15, and 18 days, respectively. Finally, larvae in the sixth treatment were denied food for the duration of the experiment. Following the starvation period, larvae in feeding treatments were offered live brine shrimp nauplii (*Artemia* spp.) *ad libitum* twice daily for the duration of the experiment. Prior to daily feedings, a 100 mL volumetric pipette was used to remove all uneaten nauplii and wastewater so as not to excessively disturb larvae. Following this cleaning procedure, 600 mL of de-chlorinated water (7.0–8.0 mg/L of dissolved oxygen) was replaced using the same pipette. Trays for the starved replicates were also cleaned and water replaced in order to maintain similar methods of handling for each treatment. All trays were held in a water table (75 cm x 150 cm) kept at a constant 17°C. A fluorescent light (photoperiod set at 15 h light/9 h dark) placed above the experimental table provided a light intensity of ~700 lux at the water surface (Lutron LX-101 lux meter; intensity chosen based on Richmond and Kynard [37]).

Larval shortnose sturgeon have been reported to be more active at night than during daylight hours directly after hatching [37]. However, the diel activity pattern through ontogeny of our larvae showed equal activity levels in separate light treatments as in dark treatments at the time of yolk absorption (Litvak and Hardy, unpublished data). Therefore, we chose to run this experiment during daylight hours.

### Survival and PNR

The PNR for larval shortnose sturgeon was defined as the time when 50% of larvae offered *Artemia* nauplii failed to initiate feeding [12]. Incidence of feeding was assessed daily by visually examining (without handling) the gut color after ~1 h of darkness; shortnose sturgeon larvae experience a loss of body coloration, and their gut becomes semi-transparent following darkness. The cumulative number of dead larvae for each treatment replicate was calculated each day (excluding loss through sampling). All mortalities were removed each day and examined for evidence of cannibalism. Taking into consideration that larval cannibalism during the stage of transition from endogenous to exogenous feeding may have an effect on final PNR results, larval behavior and condition of dead larva was closely examined for such activity. However, this was found not to be a factor since dead larvae were removed on a regular basis.

### Growth

Mean absolute and specific growth rates (AGR and SGR, respectively) were evaluated by measuring larval growth for the time interval between first feeding and the next sample date (i.e. 1–6, 6–11, 11–16, 16–19). An additional growth interval was also evaluated prior to termination of the experiment between days 19–23. To obtain these measurements, larvae (n = 2) were randomly sampled from each of the three replicate trays of the experiment prior to feeding. AGR and SGR were determined with the following equations [38, 39]:

$$\text{AGR}=({\text{w}}_{2}–{\text{w}}_{1})/(t_{2}–t_{1})$$

$$\text{SGR}=100\;\text{x}\;({\text{log}}_{e}\;{\text{w}}_{2}-{\text{log}}_{e}\;{\text{w}}_{1})/(t_{2}-t_{1})$$

where w_*i*_ is DW (dry weight) or SL (standard length) at time *t*_*i*_.

Because the primary objective of measuring growth was to determine if larvae that are fed following various periods of starvation have different growth rates than those fed continuously, the treatment with larvae that were never fed were excluded from this evaluation.

To calculate growth using SL and DW data, larvae were sampled at 1, 6, 11, 16, 19, and 23 days post-yolk absorption (or 16, 21, 26, 31, 34, and 38 dph, respectively) following Hardy and Litvak [40]. In brief, each larva was anaesthetized with 25 mg/L solution of tricaine methanesulfonate (MS-222), placed under a dissecting microscope (Olympus SZ6045), and imaged for later analysis. SL (mm) from the anterior most point of the developing rostrum to the posterior most point of the notochord was measured with an image analysis system (Optimas v5.2 BioScan Inc., Edmonds, Washington). Larvae were then preserved in 10% phosphate-buffered formalin for later DW analysis. Larval DW was obtained by placing preserved larvae into pre-weighed aluminum foil containers and then into a drying oven for 24 h at 60°C. Dried larvae were then weighed to the nearest 0.0001 g on an electronic micro-balance (Mettler AE 240).

### Swimming activity and speeds

Larval swimming behavior and activity levels were recorded with a video camera (Panasonic WVDB 400) placed over the top of each individual rearing tray. The video signal was passed through a time date generator (Panasonic WJ-810) before being recorded so that each frame would be timed to the nearest 1/100 of a second. The camera, attached to a sliding apparatus, was moved into place over each individual rearing tank with minimal disturbance from vibrations. Larval activity was recorded for 20 s per replicate at a speed of 30 frames/s at a shutter speed of 500 frames/s. In order to minimize disturbance from camera manipulation as well as the tactile and vibration stimulation used to elicit escape responses, all recordings were only performed at the beginning of each delayed feeding treatment at 1, 6, 11, 16, 19, and 23 days post-yolk absorption. The recordings occurred prior to the first feeding of that particular sample day and after the first removal of tank mortalities and waste. A 5 min acclimation period [41] was then allowed following the cleaning to reduce any influence on swimming behavior. From these recordings, the proportion of larvae actively swimming or not swimming (body movements with no forward motion or not moving at all) was calculated. In addition to activity levels, swimming performance was also analyzed using the image analysis system. Swimming data were obtained only from larvae that were actively swimming within the 20 s observation period. These larvae were assigned numbers and three were randomly chosen for analysis of swimming performance. Average speed of each swimming bout was recorded. Coordinate points on swimming performance were obtained for each larva every second of the recorded swimming bout.

### Escape response and speeds

Two types of predatory attack simulations, tactile and mechanical vibration, were used to examine escape behavior and responsiveness of shortnose sturgeon larvae. These two types of stimulations may be received by very different sensory pathways. For example, a tactile response may be received by free neuromasts along the body wall, while a vibration through the water column may be received by the otic bulla and gas filled swim bladder [42]. Some sturgeon species, such as Sevruga sturgeon, *A*. *stellatus* larvae are reported to develop their lateral line canals at some point after hatching yet at hatch possess many free neuromasts around the body with aggregations along the lateral portion of the body wall [43]. Scanning electron micrographs of shortnose sturgeon larvae incubated at 13–15°C began forming their lateral line at 9 dph [37]. Shortnose sturgeon respond to a vibration with a typical “c-type” startle response [40].

To measure the response to these two types of stimuli, each replicate tray was placed in a 3-walled experimental chamber (to reduce visual stimulation) with the top open for video recording and one side open to perform the experiment. Both tactile and mechanical vibration response measurements were obtained on the same days that swimming activity and growth were measured (at 1, 6, 11, 16, 19, and 23 days post-yolk absorption).

### Tactile stimulation

Similar to swimming activity, the tactile stimulation measurements were also taken prior to that days first feeding but following the removal of mortalities and waste. After the individual replicate tray was placed into the 3-walled chamber for the stimulation treatment, a 5-min acclimation period was given to allow fish to return to baseline movements. Following this acclimation, the first escape response was induced by tactile stimuli by touching the posterior trunk of the larvae with a small blunt probe and recording the subsequent reaction. Three randomly chosen fish from each replicate were “attacked” with the probe. Care was taken to eliminate any larvae from the random selection process that were bumped into by larvae that were previously disturbed. In order to avoid repeated stimulation (reducing confounding affects from habituation) each larva “attacked” was only stimulated once by the probe during a trial. The subsequent reaction was recorded at a speed of 30 frames/s (shutter speed of 500 frames/s) with a video camera over the experimental chamber, attached to a time date generator (Panasonic WJ-810). Coordinate points on escape responses were obtained for each larva at every other frame (1/15th s) of recorded response. Mean and maximum (“burst”) escape speeds, which allows larvae to escape attacks by lunging predators, were determined using these data.

### Mechanical vibration

Following the tactile response stimulation, another 5-min acclimation period was provided to allow larvae time to return to their baseline activity. The second predatory attack, simulating vibration disturbances from attacking predators, was achieved by releasing a rubber ball (8.80 g) attached to a 24 cm long string which swung from a pre-measured height of 26 cm. After the ball was released, it struck the side of the rearing tray sending a vibration through the water column. Again, in order to avoid repeated stimulation, the entire tray was only stimulated once following isolation from other surrounding trays. The reaction was recorded with the system described above. We determined the proportion of larvae responding to the vibrations caused by the rubber ball, indicated by “C-type” body movement (a rapid escape movement of the body trunk in the shape of a ‘C’ [42]) from the video. Due to video problems, the swimming and escape activity of only one replicate tray was properly video recorded at the start of the experiment (SL and DW were all obtained for each treatment). To obtain this missing swimming and escape activity, more larval shortnose sturgeon were obtained from Canadian Caviar. The experiment was repeated at yolk absorption for all treatments. To account for possible differences in larvae due to year and parentage, SL and DW of larvae were compared with the previous year. The comparison of day 1 post-yolk absorption data showed no significant difference in SL and DW at the start of the experiment (*F*_(1,40)_ = 2.26, *P* = 0.14 and *F*_(1,40)_ = 0.87, *P* = 0.36, respectively). In addition, mean swimming and escape activity of the one replicate video recording at the beginning of the experiment were all found to be within the range of the new day 1 post-yolk absorption replicates. Therefore, swimming and escape data of shortnose sturgeon from the additional larvae were substituted at the start of the experiment.

### Statistical analysis

We analyzed growth (SL and DW) data, swimming speed and activity data, as well as escape speed (max and mean) and escape response data using linear mixed effects models with a restricted maximum likelihood (REML) approach for parameter estimation (Mixed Model procedure). We included sampling time and treatment as fixed factors in our models as well as a time × treatment interaction term. To account for the repeated measures structure of our data, we included tank (the subject or replicate) as a random effect and employed an autoregressive covariance structure [AR(1)] [44]. When a significant interaction between main effects was present, the model was separated into one-way ANOVAs (Proc ANOVA; SAS Institute [45]) to reveal significance between treatments at each sampling day. Least–square means (LSMs) were used for *a posteriori* comparisons, and probabilities were adjusted for multiple comparisons using Tukey’s correction [45]. We compared measurements (SL and DW) of the larvae at yolk-absorption between treatments using one-way ANOVAs to confirm no significant differences at the start of the experiment. We also used one-way ANOVAs to assess differences among treatment groups in absolute and specific growth rates, survival, and PNR. Mean absolute and specific growth rates of SL and DW were compared among treatments for the interval directly following first feeding and prior to the termination of the experiment. We examined survival to first feeding and survival to end of the experiment. Finally, PNR was assessed by comparing mean percent of larvae feeding after 1 h of their first exposure to food. Level of significance for main effects was set at an α of 0.05. All data were tested for normality and homogeneity of variance (Proc Univariate: SAS Institute [45]; Fmax test [46]). Analyses were run on log_10_ transformed data in cases of non-normality or heterogeneity of variance. Percentage data was arcsine-square-root transformed to meet the assumptions of ANOVA.

### Ethics statement

This study was carried out in strict accordance with the recommendations in the Canadian Council Animal Care (CCAC) guidelines on the care and use of fish in research, teaching, and testing and were approved by the University of New Brunswick Animal Care Committee. Embryos were provided by Canadian Caviar and were incubated and hatched at the University of New Brunswick. Shortnose sturgeon are a species of special concern but not endangered in Canada [28].

#### Scientific justification for experimental design

To determine the PNR and predatory escape response in the early life of shortnose sturgeon, various levels of starvation and subsequent mortality of treatment replicates were required as an endpoint for this research. Early-life mortality in sturgeon is not well researched or understood. Yet this stage is particularly important in determining year-class strength. Therefore, research on effects of low food availability is paramount in areas of excessive habitat loss and population decline. As indicated above, the 23-day experiment consisted of six delayed feeding treatments of 0, 5, 10, 15, 18, and 23 days (starved) of delayed feeding. Each treatment contained three replicate tanks with 30 larvae in each for a total of 450 larvae in the feeding trials. At the end of the 23 day experiment, 22% of larval fish (100) mortalities were recorded due to starvation. An additional 12 larvae across all treatments were sampled every 5 days to determine somatic growth during the experiment. To do this, each larva was first anaesthetized with 25 mg/L solution of tricaine methanesulfonate (MS-222), then placed under a dissecting microscope and digitally imaged for later analysis. These larvae (n = 60) were then euthanized with dose and duration of MS-222 (500 mg/L for 10 min) consistent with CCAC guidelines for Fish Care [47]. Larvae were then preserved in 10% phosphate-buffered formalin for later DW analysis. Once the research was concluded, all remaining larvae were returned to Canadian Caviar where the broodstock were held.

## Results

### Growth

**Fig 1:**
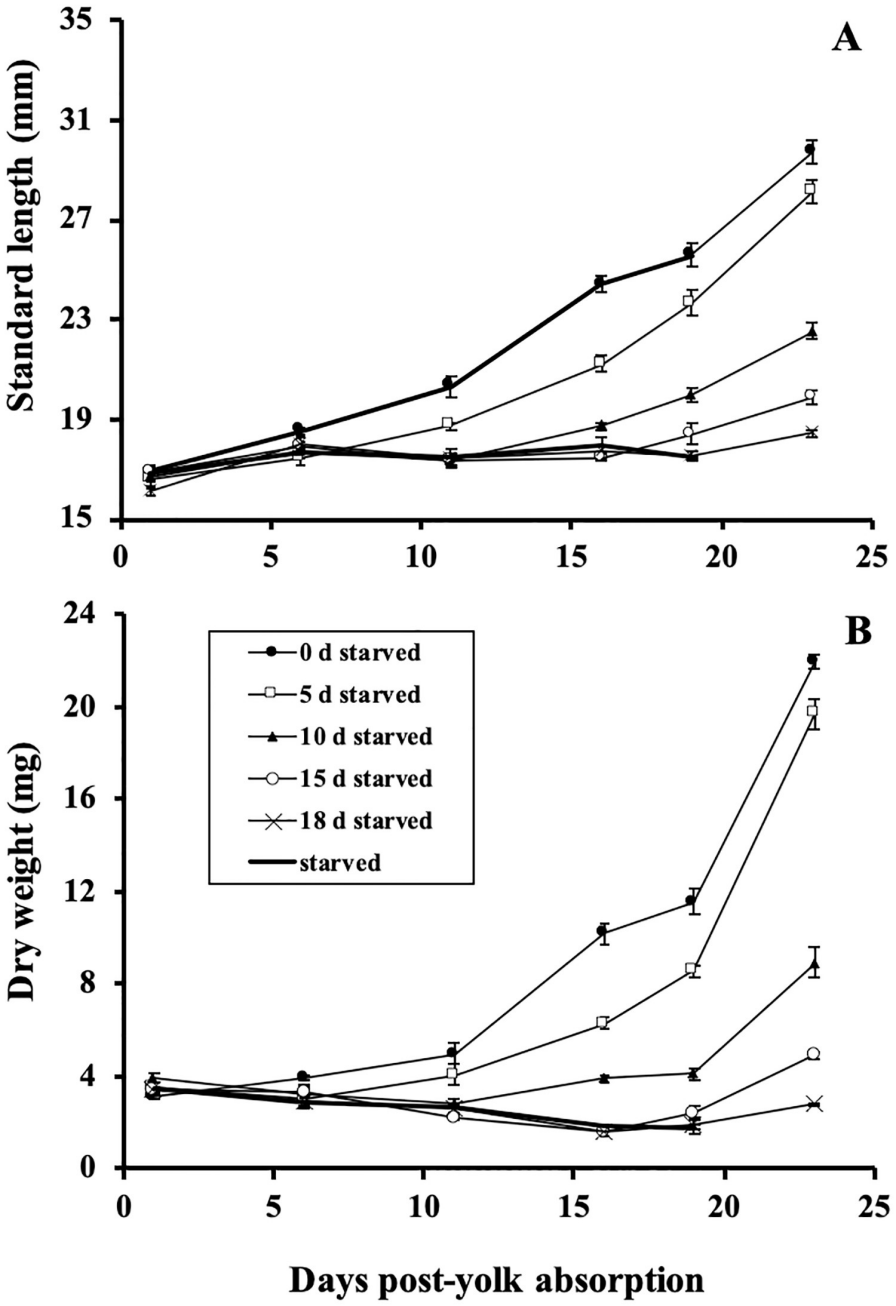
Mean standard length (A) and dry weight (B) for larval shortnose sturgeon, *Acipenser brevirostrum* in each delayed feeding treatment. Error bars represent ± 1 standard error.

SL and DW were not significantly different between the delayed feeding treatments at the start of the experiment (Fig 1A and 1B). Linear mixed effects models on mean SL and DW between these treatments over the experimental period showed interactions between the delayed feeding treatments and sample time (*F*_(24, 41)_ = 30.4, *P* < 0.001 and *F*_(24, 58.5)_ = 19.6, *P* < 0.001, respectively) and that growth in SL and DW were delayed with increasing delayed feeding (Fig 1A and 1B; Table 1). Mean AGR and SGR of SL and DW (with the exception of SGRs of DW) directly after food introduction showed no notable differences between the delayed feeding treatments (Table 1). Mean SGR of DW was higher for larvae that received 15 days of delayed feeding, compared to the 0, 5, and 10 days of delayed feeding treatments (Table 1). However, mean growth rates of SL and DW for the period at the end of the experiment (19 to 23 days post-yolk absorption) were generally higher in the delayed feeding treatments fed for longer periods of time. During this interval, mean AGR and SGR were highest for SL and DW in treatments denied food for 5 and 10 days respectively (Table 1).

**Table 1:** Mean absolute and specific growth rates (AGR and SGR, respectively) for the interval directly following first feeding (i.e. 1–6, 6–11, 11–16, and 16–19 days post-yolk absorption) and prior to the termination of the delayed feeding experiment (19 to 23 days post-yolk absorption).

Growth interval (days)	Treatment groups (days of delayed feeding)	Mean SL AGR (mm/day)	SE	Mean DW AGR (mg/day)	SE	Mean SL SGR (%/day)	SE	Mean DW SGR (%/day)	SE
Beginning feeding									
1–6	0	0.322 ^A^	0.032	0.160 ^A^	0.020	1.841 ^A^	0.188	4.558 ^A^	0.601
6–11	5	0.264 ^A^	0.016	0.214 ^A^	0.014	1.462 ^A^	0.101	6.242 ^A^	0.643
11–16	10	0.273 ^A^	0.031	0.213 ^A^	0.018	1.516 ^A^	0.177	6.461 ^A^	0.754
16–19	15	0.225 ^A^	0.010	0.267 ^A^	0.019	1.270 ^A^	0.054	13.608 ^B^	1.672
19–23	18	0.231 ^A^	0.024	0.233 ^A^	0.051	1.284 ^A^	0.128	10.197 ^AB^	2.564
End feeding									
19–23	0	1.125 ^A^	0.109	2.541 ^A^	0.151	4.060 ^A^	0.427	15.574 ^AB^	1.065
19–23	5	1.117 ^A^	0.081	2.781 ^A^	0.122	4.324 ^A^	0.335	20.803 ^A^	0.548
19–23	10	0.638 ^B^	0.028	1.208 ^B^	0.110	3.005 ^AB^	0.126	19.429 ^A^	0.749
19–23	15	0.448 ^BC^	0.062	0.625 ^C^	0.080	2.356 ^BC^	0.315	17.882 ^AB^	2.758
19–23	18	0.231 ^C^	0.024	0.233 ^C^	0.051	1.284 ^C^	0.128	10.197 ^B^	2.564

Mean values represent replicate trays within each treatment (n = 3). Significance between treatment means is indicated by different superscript letters (Proc GLM [42]). SL = standard length; DW = dry weight; SE = standard error.

### Survival and PNR

**Fig 2:**
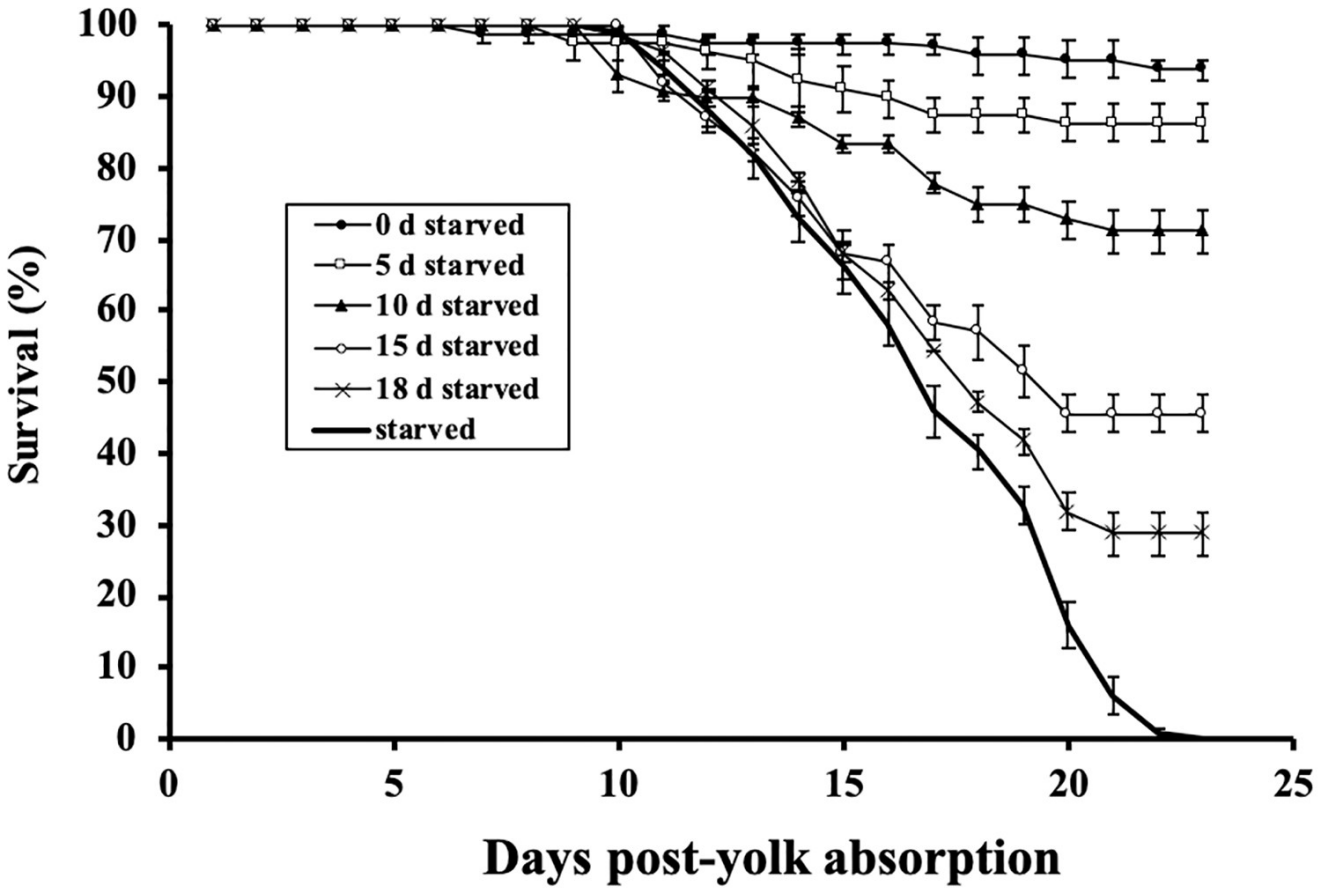
Mean survival for shortnose sturgeon, *Acipenser brevirostrum* in delayed feeding treatments. Error bars represent ± 1 standard error.

Treatments denied food for 0 and 5 days exhibited 100% survival up to the time food was offered. However, there were substantial declines on survival to first feeding in the more intense starvation treatments (*F*_(4,10)_ = 339.9, *P* < 0.001). Survival (± SE) for larvae experiencing delayed feeding for 10, 15, and 18 days dropped to 90.5% ± 1.19, 66.7% ± 2.56, and 41.7% ± 1.83, respectively (*P* < 0.001; Fig 2). Survival to the end of the experiment also varied among treatment groups (*F*_(4,10)_ = 108.5, *P* < 0.001). There was no difference between the 0 and 5 days delayed feeding treatments, however survival to the end of the experiment in treatments with delayed feeding for 10, 15, and 18 days was again reduced to 71.2% ± 3.03, 45.4% ± 2.62, and 28.8% ± 3.03, respectively (*P* < 0.001; Fig 2). Replicate tanks from the starved treatment, showed 100% mortality by 22 days post-yolk absorption (37 dph).

Shortnose sturgeon had a PNR (55.7% initiated feeding) at ~19 days following the full absorption of the yolk (42 days post-fertilization; 34 dph) at 17°C. Feeding success (%) was similar between the 0 and 5 delayed feeding treatments, while feeding in the 10, 15, and 18 day delayed feeding treatments was greatly reduced (Table 2).

**Table 2:** Results from ANOVA on the effect of delayed feeding on the mean percent of shortnose sturgeon, *Acipenser brevirostrum* larvae feeding after 1 hour of their first exposure to food.

Treatment (days of delayed feeding)	Days-post yolk absorption (sampling day)	Age (day post- hatch)	Age (day post-fertilization)	% Feeding (un-transformed data)	SE
0	1	16	24	100.00 ^A^	0.000
5	6	21	29	100.00 ^A^	0.000
10	11	26	34	90.26 ^B^	1.174
15	16	31	39	71.46 ^C^	2.487
18	19	34	42	55.71 ^D^	2.974

Analysis was run on arcsine square-root transformed data to meet assumptions of normality. Mean values represent replicate trays within each treatment (n = 3). Significance between treatment means is indicated by different superscript letters. SE = standard error.

### Swimming activity

**Fig 3:**
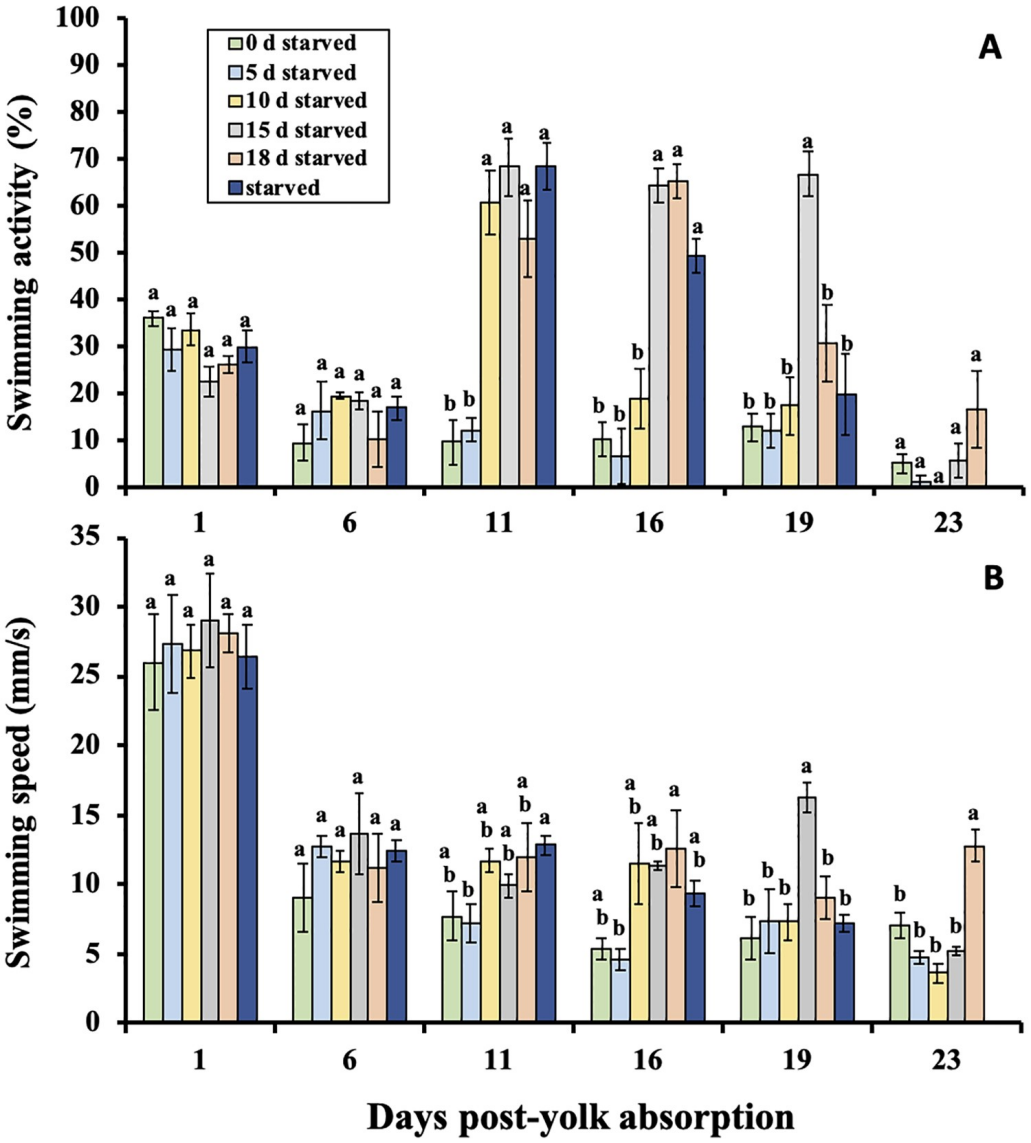
A) Mean percent of larval shortnose sturgeon, *Acipenser brevirostrum* in each delayed feeding treatment actively swimming and B) mean swimming speeds. Groups with different letters are significantly different (*P* < 0.05) at that sample period. Error bars represent ± 1 standard error.

There was an interaction between delayed feeding treatments and sample time (*F*_(24,48.9)_ = 5.90, *P* < 0.001). At 1, 6, and 23 days post-yolk absorption no differences in swimming activity were detected between the delayed feeding treatments (Fig 3A). However, at 11 and 16 days post-yolk absorption the percent of larvae swimming generally increased as delayed feeding increased, while at 19 days the swimming activity followed a dome-shaped function (Fig 3A).

### Swimming speeds

Mean swimming speeds over the experimental period showed an interaction between delayed feeding treatments and sample time (*F*_(24, 56.3)_ = 3.19, *P* < 0.001). It is interesting to note that swimming speeds decreased from 1 to 6 days post-yolk absorption for all treatments (Fig 3B). Resulting one-way ANOVAs at each sample day showed no significant difference in swimming speeds between delayed feeding treatments on 1 and 6 days post-yolk absorption. Larvae in the 5 day delayed feeding treatment had significantly slower swimming speeds at 11 and 16 days post-yolk absorption (Fig 3B). Differences among treatment groups also occurred on day 23 post-yolk absorption (*F*_(4,8)_ = 19.6, *P* < 0.001), where the 18 day delayed feeding treatment showed significantly higher swimming speeds than all other fed treatments (Fig 3B).

### Escape response to probe

**Fig 4:**
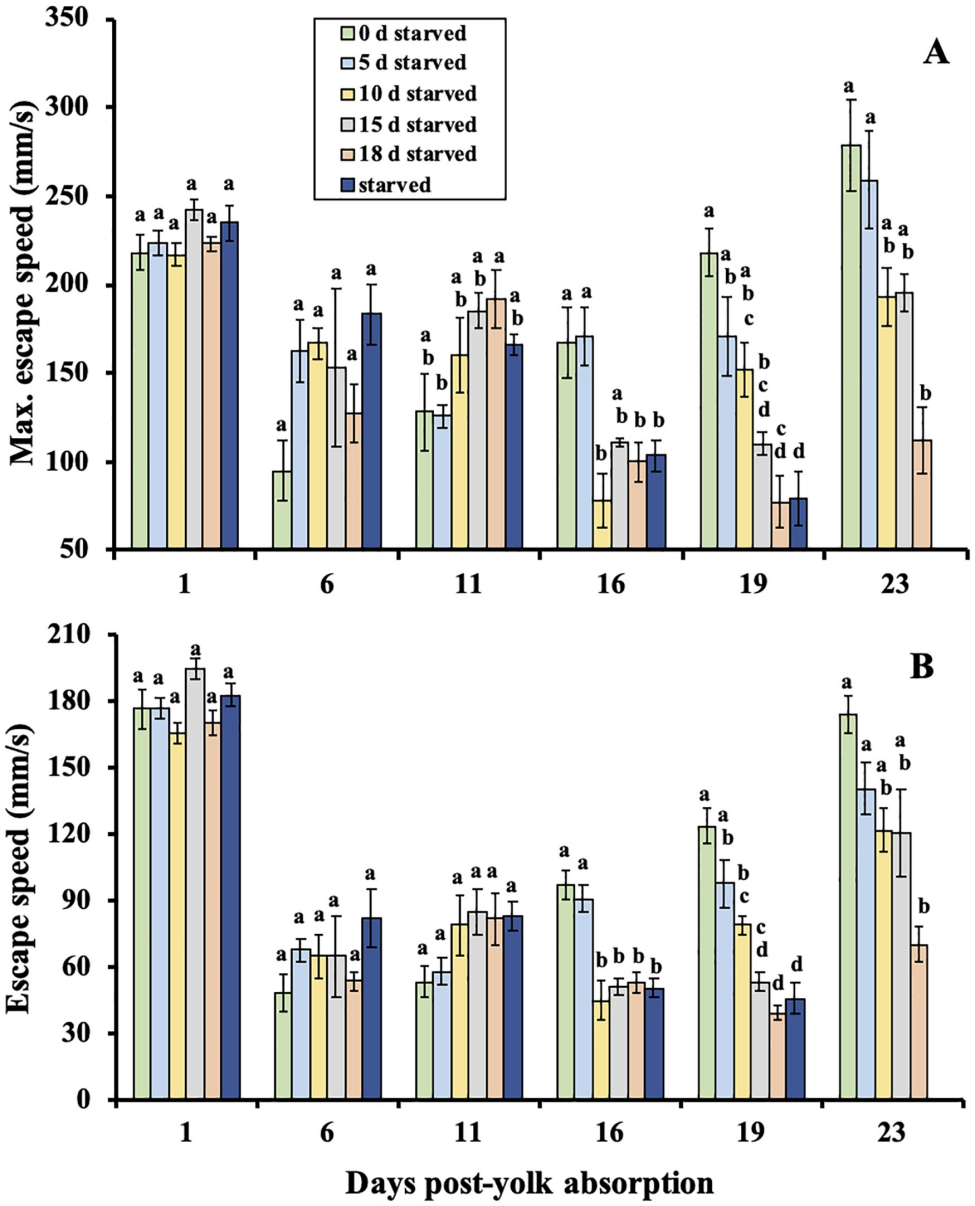
Maximum escape speeds (A) and mean escape speeds (B) for larval shortnose sturgeon, *Acipenser brevirostrum* in each delayed feeding treatment. Groups with different letters are significantly different (*P* < 0.05) at that sample period. Error bars represent ± 1 standard error.

There was an interaction between delayed feeding treatments and sample time for mean and maximum escape speeds (*F*_(24, 54.3)_ = 5.72, *P* < 0.001 and *F*_(24, 54.9)_ = 6.33, *P* < 0.001, respectively; Fig 4A and 4B) over the experimental period. At 1 and 6 days post-yolk absorption there were no differences in maximum escape speed between the delayed feeding treatments (Fig 4A). By day 16 post-yolk absorption, maximum escape speeds were typically decreased for larvae that were denied feeding for longer periods of time (≥ 10 days of delayed feeding). No differences in mean escape speeds were detected between delayed feeding treatments on 1, 6, and 11 days post-yolk absorption. For the remaining days, larvae that were denied feeding for longer periods of time had slower overall escape speeds (Fig 4B).

### Escape response to mechanical vibration

**Fig 5:**
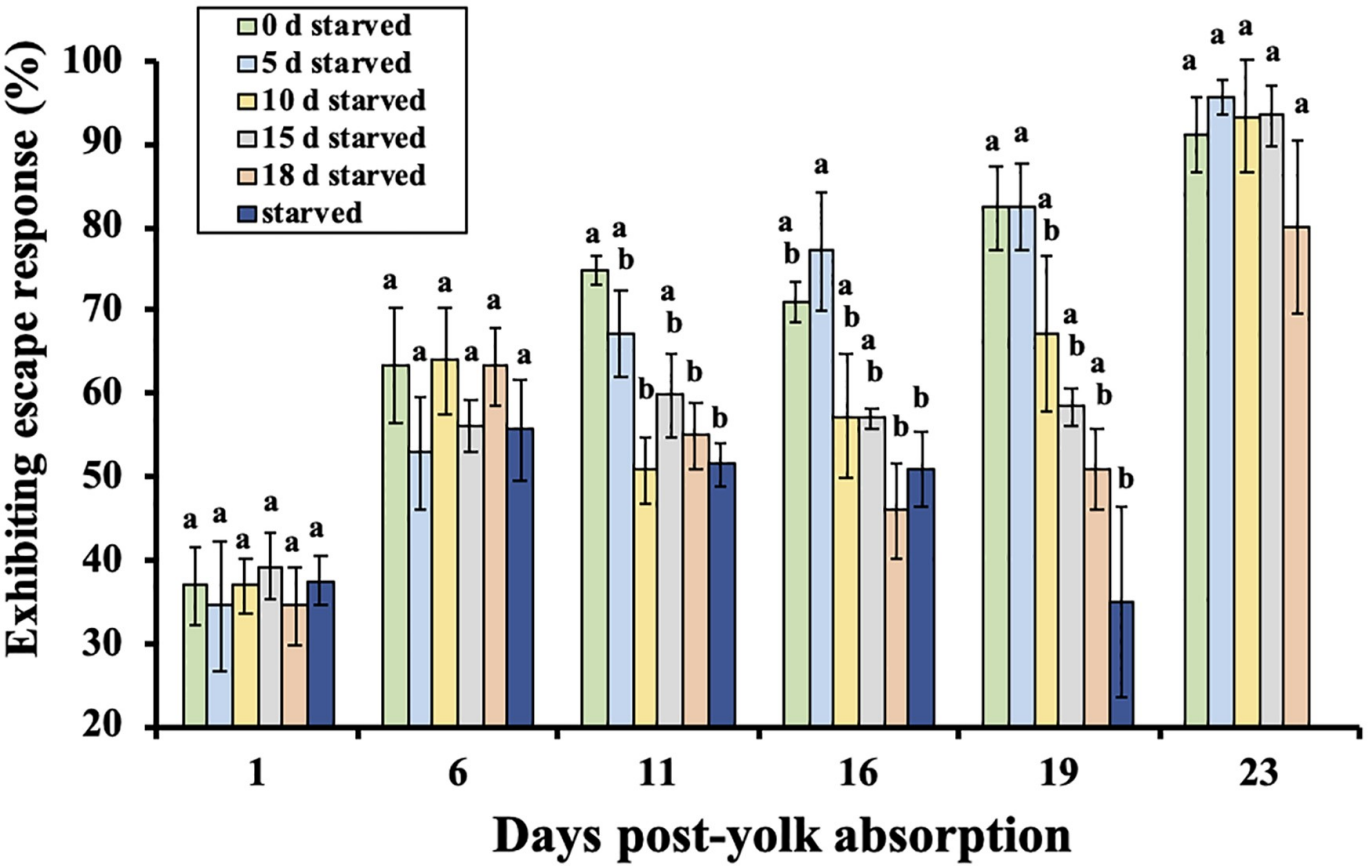
Mean percent of larval shortnose sturgeon, *Acipenser brevirostrum* in each feeding treatment exhibiting escape response (to ball striking side of tank). Groups with different letters are significantly different (*P* < 0.05) at that sample period. Error bars represent ± 1 standard error.

All treatments exhibited a positive escape response from 1 to 23 days post-yolk absorption (Fig 5). The percent responding to the mechanical vibration increased with level of feeding, and those denied food tended to exhibit less of a response until food was provided (Fig 5). There were no notable changes in percent response on 1, 6, or 23 days post-yolk absorption, but such changes were observed among groups on the remaining days (day 11: *F*_(5, 15)_ = 6.80, *P* = 0.002; day 16: *F*_(5, 15)_ = 4.85, *P* = 0.008; day 19: *F*_(5, 15)_ = 4.57, *P* = 0.01).

## Discussion

We found that starvation affected growth and survival, yet despite the degree of starvation, larvae were able to resume growth and experience high survivorship following feeding. Shortnose sturgeon larvae also adjusted strategies in behavior relative to their degree of starvation. Since starvation causes the arrest of normal tissue synthesis [48], a delay in growth with increasing degrees of starvation is to be expected. For larval shortnose sturgeon, the resumption of normal growth, despite the degree of starvation, may allow a rapid recovery and restoration of damaged tissues that may have occurred during this period. This type of recovery growth has also been reported in delayed feeding studies of Siberian sturgeon, *A*. *baeri* [49], Chinese sturgeon, *A*. *sinensis* [50], Persian sturgeon, *A*. *persicus* [51], as well as larvae of more modern teleost species like gilthead seabream, *Sparus aurata* [19], walleye pollock, *Gadus chalcogrammus* [20], and silver therapon, *Leiopotherapon plumbeus* [52]. The growth “spurt” seen in larvae starved for the longest periods (15 and 18 days) may also reflect a compensatory mechanism used by these larvae when food is limited or patchily distributed. Similar short durations of compensatory growth have been reported for other fish species [53–56]. Alternatively, it may simply be an artifact of feeding rate, where those denied food for the longest term experience a short period of hyperphagia, defined as the excessive ingestion of food beyond that needed for basic energy requirements [57, 58].

Little information exists as to when larval sturgeon should begin feeding following the absorption of endogenous energy reserves [49, 59]. Our study indicates that shortnose sturgeon larvae can survive for a remarkable ~42 days post-fertilization (19 days post-yolk absorption) at 17°C to the point of irreversible starvation. Comparing shortnose sturgeon larvae to the PNR of 25 marine species summarized by McGurk [60], shortnose sturgeon take considerably longer to reach PNR from fertilization than most species with similar ELH stages. Our observed PNR of 42 days post-fertilization was significantly longer than the predicted PNR of 30.7 days post-fertilization ± 0.43 SE by McGurk’s original regression equation (t_s_ = 0.5 + 1.3*t_y_; where t_s_ is the time from fertilization to PNR and t_y_ to yolk absorption). Like shortnose sturgeon, larval bloch, *Channa striatus* (a freshwater species) also had a PNR [61] well above that predicted by McGurk’s regression equation. Shan et al. [62] used degree-days to compare PNR across species. Compared to their list of aquatic species, shortnose sturgeon, at 578 degree-days, was more than double the highest PNR reported in their study. Shortnose sturgeon have larger eggs and a much longer time to PNR compared to the 25 species analyzed by McGurk [60]. Lengthened time to PNR may be related to the presence of a large oil globule within the eggs of shortnose larvae similar to some other fish larvae [60–63]. The oil globule (which was 45% ± 0.03 SE of the larval shortnose sturgeon total yolk volume at hatch) is absorbed more slowly than yolk during food deprivation and may contain higher energy than yolk proteins [64, 65]. Thus, it may aid in prolonging or even avoiding PNR [36, 60, 61, 64, 66]. Alternatively, this variation may reflect differences in the quality of yolk constituents as a result of differences in feeding habit and selective pressures, between freshwater and marine fish.

Larval shortnose sturgeon experienced a reduction in swimming and escape speeds from 1 to 6 days post-yolk absorption. This may have been caused in part by a physiological or behavioral development, such as a switch from cutaneous to gill respiration occurring near the yolk absorption stage. The switch to gill respiration may result in better oxygen supply to tissues and ultimately a change in swimming behavior and activity patterns [67, 68]. Swimming and escape velocities are very important in determining the outcome of a predatory attack [6, 69]. It has been suggested that sturgeon are relatively slow swimmers and possess poor acceleration ability [70, 71]. This assumption does not seem to be the case for shortnose sturgeon during the larval stage. Shortnose sturgeon larvae in the non-delayed feeding group exhibited swimming speeds comparable to that of other fishes. For example, larval shortnose sturgeon exhibited mean swimming speeds of 1.4 BL (body lengths)∙s^-1^, which is similar to speeds of 0.9–1.2 BL∙s^-1^ reported for larval striped bass, *Morone saxatalis* [72] and swimming speed of 1.5 BL∙s^-1^ for larval bloater, *Coregonus hoyi* [73]. Shortnose sturgeon also exhibited similar escape speeds when compared to other species. For example, in non-delayed feeding treatments, larval shortnose sturgeon had mean and maximum escape speeds as high as 11.4 BL∙s^-1^ and 19.4 BL∙s^-1^, respectively. Other studies have reported similar ranges of mean and maximum escape speeds such as 5.7–8.6 BL∙s^-1^ and 12.1–16.1 BL∙s^-1^, respectively, for several marine fish species [7] and 5.9–15.0 BL∙s^-1^ for American plaice, *Hippoglossiodes platessoides* [74] (all in response to pipette or tactile stimulation). Since sturgeon live most of their lives in swift current environments, the ability to successfully swim, accelerate, and maintain position in swift river environments may require the use of such locomotor abilities.

Here, we proposed 4 couplets of potential responses to predation in response to starvation. Based on the responses observed, arguments can be made in support of both couplets 2 and 4. Similar to the second hypothesized behavioral response identified in our objectives, larval shortnose sturgeon denied food during this experiment typically responded by increasing their swimming speeds and activity with increasing levels of starvation while the proportion of larvae responding to an attack were generally similar until PNR. Our results contrast findings that some larval fish reduce their activity levels during starvation [72, 75] to conserve energy. In some other species like Atlantic cod, *Gadus morhua*, it was shown that swimming activity was influenced by nutritional status, but the effect depended on age [76]. Other studies, however, have reported very similar larval behaviors to what we observed with shortnose sturgeon, showing an increase in swimming or “search” activity during progressive starvation until PNR is reached [7, 77]. A larva’s increased activity level combined with maintaining a certain degree of risk responsiveness may be an adaptive strategy to guard against predation attacks while increasing the means by which it locates a food patch. This, however, may increase predator encounter rates [33, 78]. Since larval swimming and escape capabilities increase with size [33, 79] the vulnerability to predation may certainly be affected by the degree of starvation. However, it is also possible that smaller and relatively slower larvae, as a result of food limitation, may be less conspicuous to predators than those exposed to higher food densities [9].

While there is support for second hypothesized response, as seen in the trend of maintenance in the percentage of larvae responding to a predatory attack prior to first feeding, the strength of their escape swimming responses seemed to decline as starvation progressed and did not rebound quickly after feeding was initiated. This was not expected, as other studies on fish larvae have reported an increase escape response directly after feeding [1, 33, 80]. In our study the delay of increased escape speeds following first feeding suggests a decrease in risk responsiveness which supports the fourth hypothesis of increased search activity and lower risk responsiveness. During the interval from starvation to satiation, metabolic rates may not re-adapt immediately to high levels of food availability [81]. In addition, newly fed larvae (starved for only short periods) may be so preoccupied in becoming satiated that they may not respond as well to a tactile stimulation.

Shortnose sturgeon larvae hatch in riverine systems, which are much different than open lake or ocean environments. Increasing activity levels during periods of starvation, especially vertically in such an environment, may allow a relocation downstream to potentially more productive areas. It would make sense that when a larva locates a suitable food patch, their swimming activity level should decrease, as was seen in the 0, 5, and 10 day delayed feeding treatments. The relatively good swimming and escape responses from the 15 and 18 day delayed feeding treatments following feeding may be strategies adopted to maximize feeding efficiency (evident by higher SGRs) and risk responsiveness once prey items are located.

## Conclusions

Since shortnose sturgeon spawn in the spring during periods of low productivity, growth may be slower and risk of starvation higher than for larvae of other species hatched during summer months. The ability of larval shortnose sturgeon to withstand relatively long periods of food deprivation past yolk absorption may be an ecological adaptation to survive periods of low food availability and patchy distribution. It is important to consider that the effects from starvation may be magnified in wild populations due to high energetic costs of negotiating large swift river systems, avoiding predation, and securing suitable nursery and foraging habitats [75, 82]. Our overall findings showed in laboratory simulations, larval shortnose sturgeon increase physical activity during periods of starvation to find a food patch while remaining vigilant but maybe not as capable to defend against a predatory attack as fed individuals.

## Supporting information

S1 File (XLSX)
